# Integrative Omics Analysis Reveals the Potential Value of CEACAM6 in Pan‐Gastrointestinal Cancers

**DOI:** 10.1002/iid3.70327

**Published:** 2026-02-04

**Authors:** Liying Jin, Changjuan Tao, Zhiyong Pan, Peng Zhang

**Affiliations:** ^1^ Department of Gastroenterology Xianju People's Hospital Taizhou China; ^2^ Department of Radiation Oncology, The Cancer Hospital of the University of Chinese Academy of Sciences Zhejiang Cancer Hospital Hangzhou China; ^3^ Department of Neurology Yueyang People's Hospital Yueyang China; ^4^ Department of Medical Oncology Zhejiang Provincial People's Hospital Hangzhou China; ^5^ Key Laboratory of Tumor Molecular Diagnosis and Individualized Medicine of Zhejiang Province, Zhejiang Provincial People's Hospital People's Hospital of Hangzhou Medical College Hangzhou China

**Keywords:** CEACAM6, pan‐gastrointestinal cancers, prognosis, tumor immune microenvironment

## Abstract

**Objective:**

Carcinoembryonic antigen cell adhesion molecule 6 (CEACAM6) is known as a cell adhesion receptor which could regulate proliferation and other signaling in cancer. The role of CEACAM6 in pan‐gastrointestinal cancers remains largely uncharacterized. This study employed multi‐omics bioinformatics to investigate the expression distribution, prognostic value, and immune function of CEACAM6 in these malignancies.

**Methods:**

Utilizing multi‐omics data from The Cancer Genome Atlas (TCGA), cBioPortal, GDSC2, TIMER2.0, and TISCH databases, we assessed the CEACAM6 expression and prognostic value across pan‐gastrointestinal cancers. Additionally, the potential role of CEACAM6 in the tumor immune microenvironment was explored using multi‐omics data, including spatial transcriptomics data.

**Results:**

Based on TCGA data, CEACAM6 was found to be overexpressed in pan‐gastrointestinal cancers. The CEACAM6 somatic copy number alterations, DNA methylation and mutation sites were identified as potential contributors to abnormal CEACAM6 expression. The CEACAM6 expression was significantly negatively associated with the abundance of CD4 + Th1 cells across pan‐gastrointestinal cancers. Furthermore, spatial transcriptomics data revealed that CEACAM6 expression was significant positively associated with malignant cells, while there was a negative correlation was observed with between CEACAM6 expression and plasma cells.

**Conclusion:**

CEACAM6 exhibits high diagnostic accuracy and tumor‐specific overexpression in pan‐gastrointestinal cancers. CEACAM6 could promote angiogenesis/metastasis and suppress anti‐tumor immunity. Spatially localized in tumors with immune cell exclusion, CEACAM6 correlates with poor survival and immune‐excluded subtypes, positioning it as a therapeutic target in precision immunotherapy for pan‐gastrointestinal cancers.

## Graphical abstract

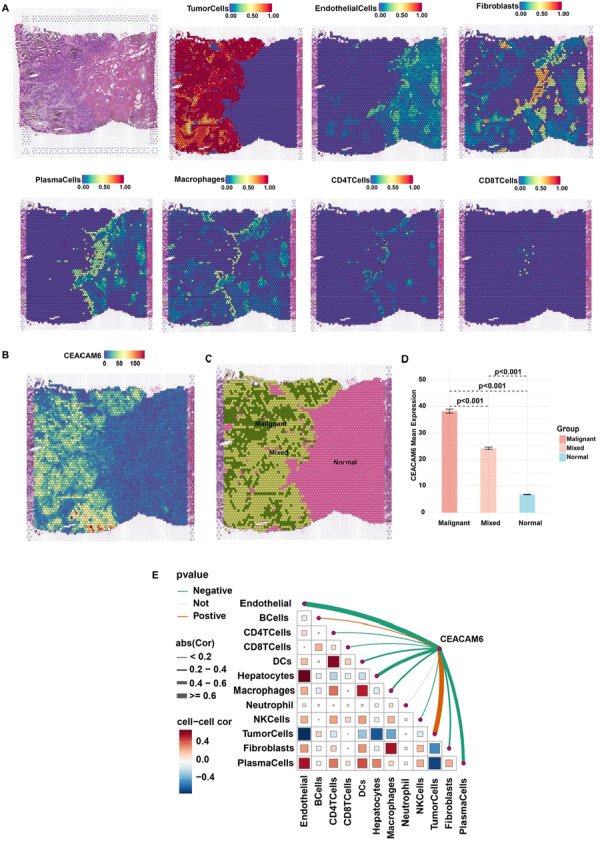

## 1 Introduction

Pan‐gastrointestinal cancers, comprising esophageal, gastric, and colorectal cancers, are among the most prevalent and fatal malignancies globally. Notably, colorectal cancer and gastric cancers rank among the top ten cancer types with the highest mortality rates worldwide [1]. In 2022, an estimated 1.9 million patients were newly diagnosed with colorectal cancer and 904,000 related deaths were reported, accounting for approximately one‐tenth of all cancer cases and deaths [1]. These pan‐gastrointestinal cancers share a common origin, arising from the columnar epithelium of the digestive system, which is derived from the endoderm [2]. Furthermore, these malignancies frequently encounter similar environmental insults that promote tumor development, resulting in the presence of shared molecular features such as aneuploidy and microsatellite instability [2]. Consequently, these pan‐gastrointestinal malignancies exhibit homogeneity and could be subclassified based on their mesenchymal gene expression profiles, metabolic characteristics and immune infiltration patterns [3]. Patients with advanced pan‐gastrointestinal malignancies often have a poor prognosis, and the efficacy of immune checkpoint inhibitors such as programmed cell death protein 1 (PD‐1) antibodies tended to be limited. Numerous studies are underway to investigate novel anti‐cancer agents, including antibody drug conjugates, traditional medicine drugs, anticancer metabolites derived from fungi and oncolytic virus [4, 5, 6, 7, 8]. There remains an urgent clinical need to identify new therapeutic targets of pan‐gastrointestinal malignancies. Integrated multi‐omics analysis of pan‐gastrointestinal cancers could reveal underlying pathogenic mechanisms, yielding critical insights into the biology of these common malignancies.

The pan‐gastrointestinal malignancies share similar endodermal developmental origins and exposure to common insults including microbial infection that promote carcinogenesis. Over 95% of pan‐gastrointestinal malignancies are classified as adenocarcinomas. This shared developmental origin, similar inflammation‐mediated carcinogenesis and uniform adenocarcinoma histology suggested the existence of common therapeutic targets across these cancers. Our prior work demonstrated that carcinoembryonic antigen cell adhesion molecule 6 (CEACAM6) was ubiquitously overexpressed across pan‐gastrointestinal cancers 14. CEACAM6 (also known as CD66c or NCA‐90) is a member of the immunoglobulin superfamily. It is a glycosylphosphatidylinositol (GPI)‐anchored cell surface protein and features an N‐terminal variable domain followed by two C2‐like immunoglobulin domains [9, 10]. CEACAM6 is GPI‐anchored and lacks both a transmembrane and cytoplasmic domain, which enables CEACAM6 to mediate cell‐cell and cell‐matrix adhesion through lateral associations with lipid rafts and co‐receptors including integrins [11]. Critically, aberrant CEACAM6 expression is an early event in pan‐gastrointestinal malignancies, detectable in precancerous lesions [12, 13]. The CEACAM6 expression escalates with disease progression, as evidenced in pancreatic intraepithelial neoplasia and gastric dysplasia, culminating in high levels in overt carcinomas [12, 13]. Functionally, CEACAM6 orchestrates multiple oncogenic processes in pan‐gastrointestinal tumorigenesis and it could promote tumor cell invasion, confer resistance to anastasis, and facilitate immune evasion and vasculogenic mimicry [14] [15] [16] [17]. Mechanistically, the co‐clustering of lipid raft‐associated CEACAM6 with integrins activates oncogenic signaling pathways, including AKT/ERK, Src‐FAK, and PI3K/AKT [15] [16] [17]. The TGF‐β/SMAD3 signaling axis transcriptionally upregulates CEACAM6, linking this pathway to the promotion of epithelial‐mesenchymal transition and tumor progression [18]. Furthermore, CEACAM6 may also be a critical modulator of the tumor immune microenvironment, through homophilic/heterophilic interactions with CEACAM family proteins. Recent study reported that CEACAM6 could suppress the anti‐tumor activity of cytotoxic T cells in vitro and promote malignant cell immune evasion by interacting with CEACAM1 [9]. The blockade of CEACAM6 could increase the production of T‐cell cytokines and effector molecules (e.g., IFNγ, IL2 and granzyme B) [9]. Consequently, CEACAM6 has been identified as a potential immune checkpoint [9]. The clinical relevance of targeting CEACAM6 is underscored by ongoing development of therapeutic agents. An antibody‐drug conjugate (ADC) targeting CEACAM6, l‐DOS47, demonstrated favorable safety at doses up to 13.55 μg/kg in a phase I/II trial [19]. A Phase I clinical trial utilizing CEACAM6 antibody in advanced solid cancer is ongoing (NCT03596372). On the other hand, glycosylation of CEACAM family members could modulate oligomerization, stability, and protease resistance, contributing to functional plasticity in tumorigenesis [20]. A new ADC which targets a tumor‐specific N256‐glycosylation site conserved on CEACAM5 and CEACAM6 (EBC‐129) recently received FDA fast‐track designation (2025).

Despite these advances, CEACAM6's spatial distribution, infiltrating immune cells correlations, and the potential transcriptional factors remain poorly characterized. These above findings motivate this study to systematically elucidate the multifaceted roles of CEACAM6 in pan‐gastrointestinal malignancies.

## 2 Materials and Methods

### 2.1 Data Acquisition and Organization

The transcriptome data with clinical features of pan‐gastrointestinal cancer including esophageal cancer (ESCA), stomach cancer (STAD), colon cancer (COAD) and rectum cancer were obtained from The Cancer Genome Atlas Program (TCGA) database and the normal tissue database (Genotype‐Tissue Expression Project [GTEx]). This study utilized normalized gene expression data from TCGA database and GTEx database. The data were transformed into unitless Z‐score values to eliminate scale discrepancies across samples. Outliers with Z‐scores below −3 or above 3 were excluded to ensure robustness. The Wilcoxon Rank Sum Test was applied to compare expression levels between tumor and normal tissues in the pan‐gastrointestinal cancer cohort. The CEACAM6 expression at the single‐cell transcript level was validated based on the data from Tumor Immune Single‐cell Hub (TISCH) database and the GEO database [21]. To evaluate the diagnostic performance of CEACAM6 in distinguishing tumor from normal tissues, receiver operating characteristic (ROC) analysis was conducted using the pROC package in R. The area under the ROC curve (AUC) and its 95% confidence interval (95% CI) were calculated, and smoothed ROC curves were generated. In addition, the protein expression levels of CEACAM6 in pan‐gastrointestinal cancer tissues and corresponding normal tissues based on the immunohistochemistry (IHC) staining were obtained from the Human Protein Atlas (HPA) database. This study adheres strictly to the data extraction policies of the databases.

### 2.2 Pathway Activity Analysis, Functional States Analysis and Visualization of PPI Network

The protein expression data of protein‐protein interaction (PPI) were obtained from The Cancer Proteome Atlas (TCPA) database, which included reverse phase protein array (RPPA) of 7876 TCGA tumor samples [22]. Pathway activity scores of 10 cancer‐related pathways (apoptosis, hormone ER, DNA damage response, hormone AR, epithelial‐mesenchymal transition [EMT], cell cycle, TSC/mTOR pathway, PI3K/AKT pathway, RAS/MAPK pathway and RTK pathways) were calculated based on the protein intensity [23, 24]. To better understand the role of CEACAM6 in pan‐gastrointestinal cancer, the Cancer SEA, which analyzed 41, 900 cancer single cells to portray a cancer single‐cell functional state atlas, was applied to explore the association between CEACAM6 and 14 functional states (EMT, differentiation, stemness, proliferation, hypoxia, invasion, angiogenesis, inflammation, apoptosis, quiescence, cell cycle, metastasis, DNA damage and DNA repair) [25]. The GSVA R package was employed to calculate a combined z‐score metric for 14 functional state gene sets using the z‐score transformation method. Subsequently, the resulting scores were further standardized and defined as gene set enrichment scores. Finally, Pearson correlation coefficients were computed to assess the associations between CEACAM6 expression levels and each of the standardized gene set scores. The functional states could reflect functional heterogeneity of malignant cells. The PPI network was constructed using ComPPI database [26]. The ComPPI, which was a cellular compartment‐specific database for PPI network visualization and analysis, could provide a reliable subcellular compartment‐based PPI database for the analysis of biological processes on the subcellular level [26]. The ComPPI database, which could introduce two scores (the interaction score and the localization score), was applied to describe the calculated probability of the data correctness, to generate the protein list that interacts with CEACAM6. The Cancer Dependency Map provided the large‐scale data of genome‐scale CRISPR‐Cas9 knockout screens in different cell lines [27]. The CRISPR‐Cas9 knockout screening results were presented as negative score. The lower negative scores indicated that the cell line would grow slower. A score cutoff of −0.5 was selected to define genes essential for cell survival.

### 2.3 Genetic Alteration Analysis, DNA Methylation Analysis, ATAC‐Seq and Motif Analysis

The mutation frequency, DNA copy‐number alterations (CNAs), mutation types and sites, and DNA methylation of CEACAM6 across the pan‐gastrointestinal cancers were calculated using the cBioPortal platform [28]. The Wilcoxon rank test was applied to compare the different methylation of *CEACAM6* between malignancies and corresponding normal tissues. The Spearman correlation was applied to analyze the difference between *CEACAM6* and promoter DNA methylation. The accessibility of chromatin for CEACAM6 was assessed by assay for transposase‐accessible chromatin with high‐throughput sequencing (ATAC‐seq). The genomic interval for ATAC‐seq was defined as ±3 kb from the transcription start site using the parameter tssRegion = c(−3000, 3000). Spearman correlation analysis investigates the relationship between methylation level and CEACAM6 expression. Bubble maps are used to visualize the differences in methylation levels of CEACAM6. The ChIPseeker package covplot function is used to visualise the genomic location of the ATAC‐seq peak of CEACAM6 in TCGA STAD cohort. A motif discovery analysis was performed on the ChIP‐seq peaks with MEME‐ChIP from the MEME Suite 5.5.5 with default settings [29].

### 2.4 Immune Infiltration Analysis, Immune Subtypes Association and Survival Analysis

We systematically characterized the correlations between CEACAM6 expression and the tumor immune microenvironment (TIME) across pan‐gastrointestinal cancers using the TIMER2.0 database [30]. The deconvolution algorithms of the immune cell infiltration included TIMER, EPIC, CIBERSORT (Based on linear support vector regression, deconvolution from microarray data, and gene expression profiling set), MCP‐counter, CIBERSORT‐abs, quanTIseq and xCell (Used to separate cell types with high correlation). The Pearman correlation was applied to test the correlation between CEACAM6 gene and different immune infiltrating cells. The deconvolution algorithms of the immune cell infiltration included TIMER, EPIC, CIBERSORT, MCP‐counter, CIBERSORT‐abs, quanTIseq and xCell. The pan‐gastrointestinal cancer patients were grouped as three subgroups according to microsatellite instability (MSI): microsatellite stability (MSS), microsatellite low instability (MSI‐L) and microsatellite high instability (MSI‐H). The Wilcoxon analysis was applied to test the differences of CEACAM6 expression among the three groups. The immune subtypes analysis was performed according to the immune landscape of cancer [31]. The immune subtypes were defined based on such macrophage markers, lymphocyte markers, the Th1‐to‐Th2 cell ratio, the extent of inter‐tumoral genetic heterogeneity, aneuploidy levels, neoantigen load, the total cellular landscape, immunomodulatory gene expression profiles, and prognostic outcomes. The six immune subtypes were C1: Wound healing subtype; C2: IFN‐γ dominant subtype; C3: Inflammatory subtype; C4: Lymphocyte depleted subtype; C5: Immunologically quiet subtype; C6: TGF‐β dominant subtype.

### 2.5 Spatial Transcriptome Analysis

Spatial transcriptomic (ST) data for one colorectal cancer patient with liver metastasis (ST‐P1) were downloaded and analyzed [32]. In order to accurately assess the cellular composition of each spot on the 10xVisium slides, deconvolution analysis was applied. Visualizations of gene spatial expression and the enrichment scores for each cell type were achieved using the “SpatialFeaturePlot” function in the Seurat package. By characterizing the cellular composition of each microregion (referred to as “spot“), we designated spots to specific cell types based on their proportional abundance. Spots with the highest proportion of malignant cells were annotated as malignant spots. To enhance comparability across sections, Z‐score normalization was applied to the expression data in R. The normalized datasets were visualized in R, enabling an intuitive representation of spatial gene expression patterns.

### 2.6 Statistical Analyses

All data were processed by statistical software package R (v. 3.5.3) or the web tools. The Pearson correlation analysis was applied to analyze normally distributed data, otherwise the Spearman correlation analysis was applied. The Wilcoxon rank sum and Kruskal–Wallis rank sum test were applied to detect the differences between multiple variables or two variables, respectively. For survival analysis, the univariate Cox regression analysis was performed to investigate the association between CEACAM6 groups with higher and lower expression levels and progression free survival (PFS) and overall survival (OS) across pan‐gastrointestinal cancers. All statistical tests were two‐tailed. A difference was considered significant if *p* value < 0.05.

## 3 Results

### 3.1 The Consistent Overexpression of CEACAM6 Across Pan‐Gastrointestinal Cancer

**Figure 1:**
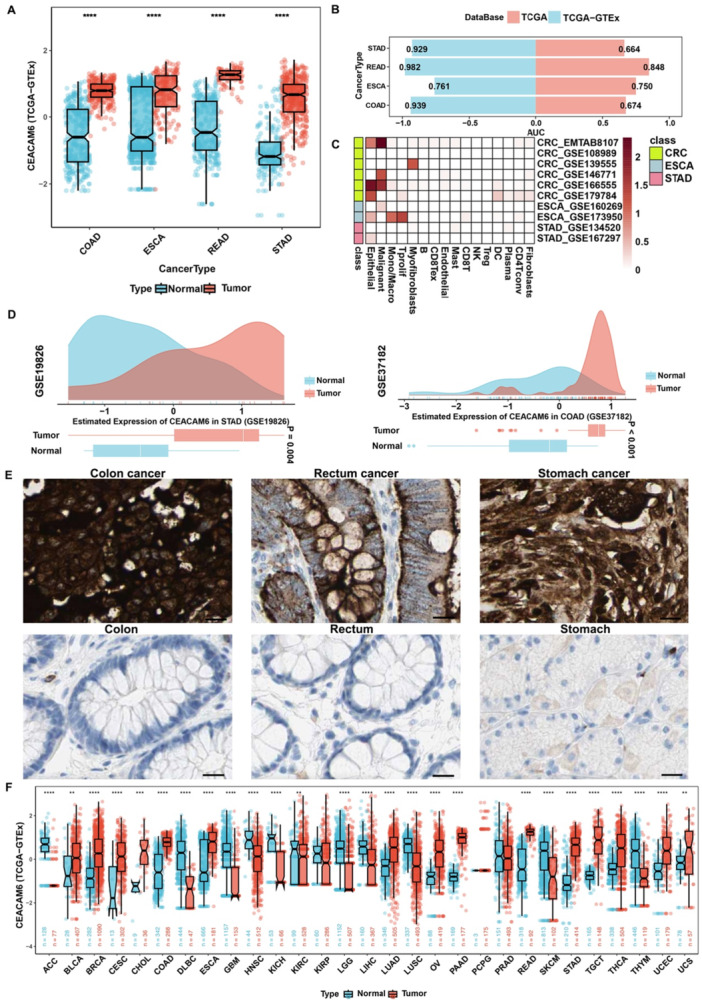
CEACAM6 was overexpressed in pan‐gastrointestinal cancer. (A) CEACAM6 transcriptional level in tumor and normal tissues across pan‐gastrointestinal cancer data of TCGA and GTEx data. ****p < 0.0001; (B) ROC curve for CEACAM6 expression to distinguish patients with malignacies from normal control. The horizontal coordinate corresponds to the AUC value of the ROC analysis; (C) The overexpression of CEACAM6 was predominantly in malignant cells at single cell level; (D) The overexpression of CEACAM6 in pan‐gastrointestinal cancers was externally validated; (E) Representative IHC images of CEACAM6 in colon cancer, rectum cancer, stomach cancer and normal tissues from HPA data; scale bar 20 μm; (F) The CEACAM6 mRNA expression among different cancer types from TCGA and GTEx database. *** *p* < 0.001; ** *p* < 0.01.

The CEACAM6 expression was firstly evaluated across pan‐gastrointestinal malignancies (COAD, ESCA, READ and STAD) and adjacent normal tissues. As shown in Figure 1A, CEACAM6 was consistently upregulated in pan‐gastrointestinal malignancies compared to adjacent normal controls. ROC curve analysis was applied to estimate the diagnostic value of CEACAM6 to discriminate malignancies. ROC analysis confirmed the high diagnostic accuracy of CEACAM6, with AUC values exceeding 0.9 in STAD, READ, and COAD cohorts (Figure 1B), underscoring its potential as a non‐invasive biomarker. Single‐cell transcriptomic data from TISCH further revealed that CEACAM6 is predominantly expressed in malignant cells, with minimal detection in immune or stromal compartments (Figure 1C), aligning with its tumor‐specific role. The Figure 1D externally validated the overexpression of CEACAM6 in pan‐gastrointestinal cancers. To validate these findings at the protein level, we analyzed IHC data from the HPA database. CEACAM6 staining showed marked overexpression in gastric, colorectal, and rectal cancers, with high scores observed in 9/12 stomach cancer cases and 10/12 colorectal cancer cases (Figure 1E). We also assessed CEACAM6 expression in the TCGA and GTEx database. As shown in Figure 1F, CEACAM6 is overexpressed in multiple malignancies, particularly pan‐gastrointestinal cancers. Collectively, these multi‐omics and cross‐platform validations established the consistent overexpression of CEACAM6 across pan‐gastrointestinal cancers.

### 3.2 CEACAM6 Regulates Cancer‐Related Pathways to Promote Tumorigenesis

**Figure 2:**
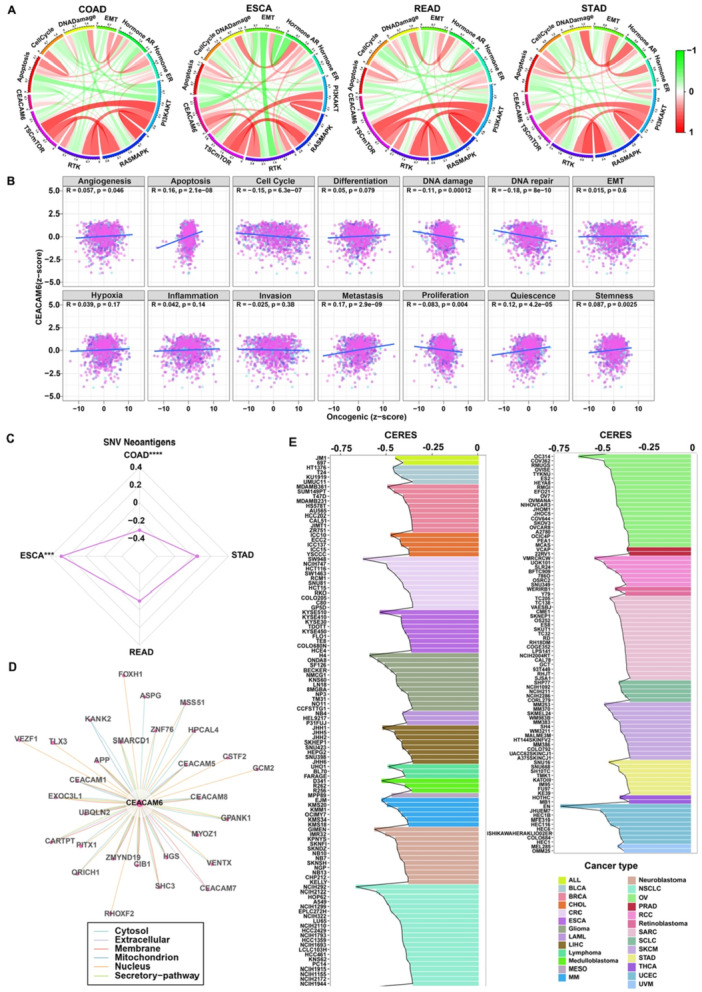
The role of CEACAM6 in tumor progression across pan‐gastrointestinal cancers. (A) The correlation of CEACAM6 expression and 10 cancer‐related pathways activity scores in COAD, ESCA, READ and STAD; (B) The Pearson correlation between CEACAM6 expression and 14 malignant features across pan‐gastrointestinal cancers; (C) Correlation of CEACAM6 expression and neoantigen expression in pan‐gastrointestinal cancers; (D) Networks of proteins interacting with CEACAM6 from comPPI database; (E) TOP200 cell lines which grow slower after knocking CEACAM6 from DepMap database.

To elucidate CEACAM6's functional roles in pan‐gastrointestinal cancers, we integrated multi‐omics data to explore its associations with key signaling pathways and cancer biology. RPPA data analysis from the TCPA database revealed that CEACAM6 expression was significantly correlated with RAS/MAPK and RTK pathway activity in COAD, ESCA, and STAD cohorts (Figure 2A), highlighting its role in oncogenic signaling. Further analysis using CancerSEA database demonstrated strong positive correlations between CEACAM6 levels and critical tumor processes, including angiogenesis (R = 0.057, *p* = 0.046), apoptosis (R = 0.16, *p* < 0.001), metastasis (R = 0.17, *p* < 0.001), and quiescence (R = 0.12, *p* < 0.001) (Figure 2B), underscoring its broad regulatory impact. Given the role of neoantigens in immune evasion and immunotherapy response, we examined their association with CEACAM6. The CEACAM6 expression showed a significant positive correlation with neoantigen load in COAD and ESCA (Figure 2C), suggesting a potential link between CEACAM6 and immune microenvironment dynamics. To further define molecular interactions, we constructed a PPI network using comPPI, which identified a strong interaction between CEACAM6 and CEACAM8 (interaction score: 0.981; Figure 2D). Functional dependency analysis via DepMap revealed that CEACAM6 knockout significantly inhibited cell growth in multiple cancer cell lines, with the top 200 most sensitive cell lines exhibiting marked growth suppression (Figure 2E). These findings collectively demonstrate CEACAM6's dual role in driving oncogenic pathways and modulating tumor‐immune interactions, while its essentiality in cancer cell survival highlights its therapeutic potential.

### 3.3 Genetic Alterations and Transcription Factor Identification of CEACAM6

**Figure 3:**
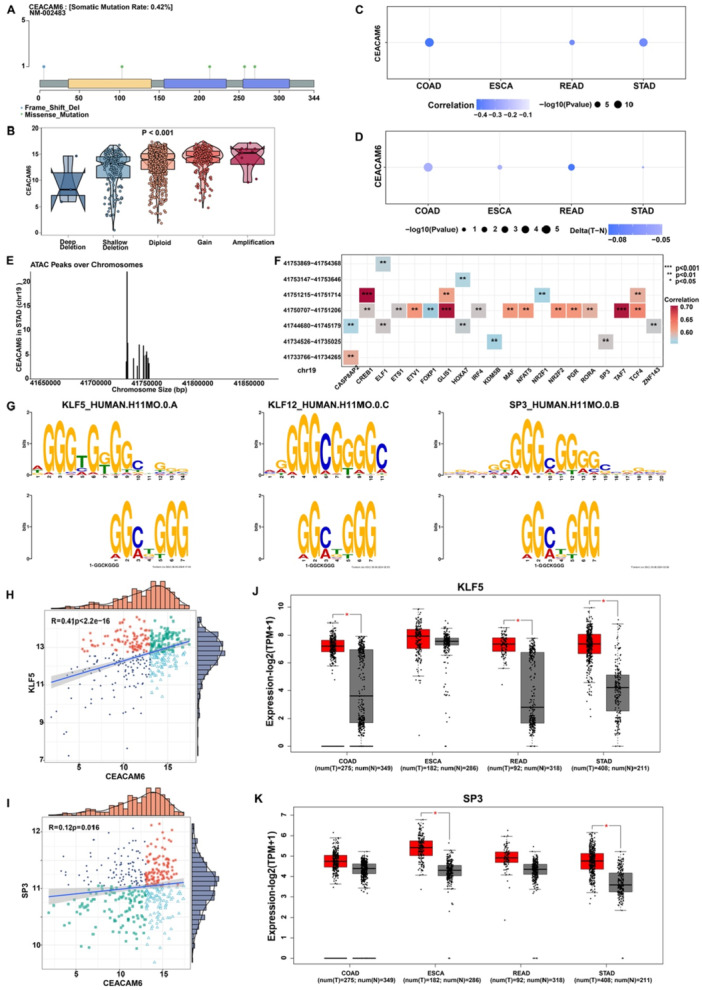
Genetic alterations and potential transcription factor of CEACAM6. (A) Sites and numbers of CEACAM6 genetic alterations in pan‐gastrointestinal cancers from cBioPortal; (B) The correlation between CEACAM6 mRNA expression and genetic alterations; (C) Spearman's correlation of CEACAM6 expression and promoter methylation level in pan‐gastrointestinal cancers. Red and blue represent positive and negative correlations, respectively; (D) The differential methylation of CEACAM6 promoter across pan‐gastrointestinal cancers in comparison with corresponding normal tissues. Hypermethylated and hypomethylated CEACAM6 are marked in red and blue, respectively; (E) The visualization of annotated ATAC‐seq peaks from the CEACAM6 promoter spanning 3000 bp upstream of the transcription initiation site to 3000 bp downstream; (F) The Spearman correlation analysis between ATAC‐peaks and annotated transcription factors; (G) The potential transcription factors binding motif identified in ChIP‐seq peaks on CEACAM6 DNA using MEME ChIP. Y axis shows frequency matrix of each base occurrence; (H‐I) The expression correlation analysis of CEACAM6 and potential transcription factors (KLF5 and SP3); (J‐K) The KLF5 and SP3 expression in tumor and normal tissues across pan‐gastrointestinal cancer data of TCGA and GTEx data.

To elucidate the molecular mechanisms driving CEACAM6 overexpression in pan‐gastrointestinal cancers, we integrated genomic and epigenomic analyses. Mutation sites of CEACAM6 in pan‐gastrointestinal cancers were visualized. In Figure 3A. The expression of CEACAM6 was up‐regulated from homozygous deletion group to high copy number amplification group (*p* < 0.001; Figure 3B). This suggests that gene dosage effects play a critical role in CEACAM6 overexpression. The Figure 3C illustrated the correlation analysis between CEACAM6 expression and CEACAM6 promoter methylation level in pan‐gastrointestinal cancers. The results revealed that the expression of CEACAM6 was significantly negatively correlated with the methylation level of CEACAM6 promoter in COAD, READ and STAD. As is shown in Figure 3D, the CEACAM6 promoter methylation level was significantly lower in cancer group.

Afterwards, we systematically identified the transcription regulator that controlled CEACAM6 expression with two methods in STAD cohort. A total of 7 ATAC‐seq peaks were annotated, which might be related to the regulation of CEACAM6 transcription, from the CEACAM6 promoter spanning 3000 bp upstream of the transcription initiation site to 3000 bp downstream (Figure 3E). Spearman correlation analysis revealed that the expression of multiple transcription factors, including KDM5B, SP3, and TCF4, was significantly associated with annotated ATAC‐seq peaks (Figure 3F). Furthermore, comprehensive motif analysis (including motif discovery) of sequences in motif location sets by MEME‐ChIP revealed the potential transcription factors including KLF5; KLF12; ZN331; ZN449; PATZ1; SP3; SP1; SP4 and SP2 (Figure 3G). The correlation analysis between potential transcription factors and CEACAM6 expression revealed that only KLF5 and SP3 was significantly positively correlated with CEACAM6 expression in STAD cohort (Figure 3H‐I). Besides, KLF5 and SP3 were both overexpressed in STAD cohort (Figure 3J‐K). To sum up, KLF5 and SP3 might be the potential transcription factor of CEACM6 in STAD cohort.

### 3.4 The Immune Role and Prognostic Significance of CEACAM6 in Pan‐Gastrointestinal Cancers

**Figure 4:**
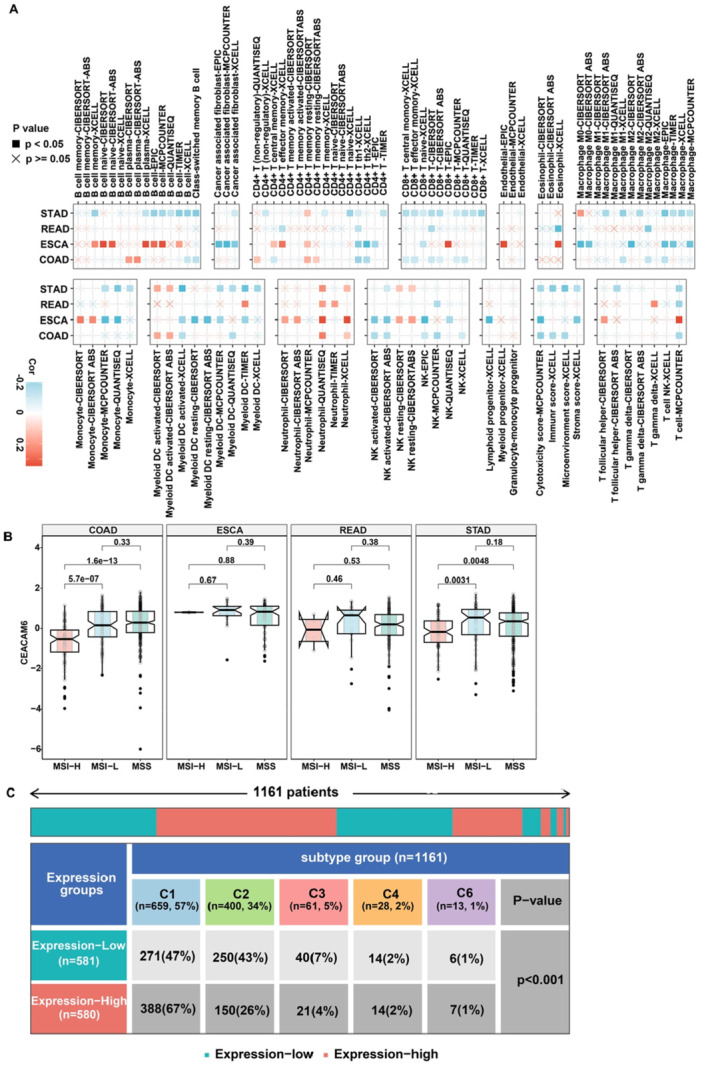
Association of CEACAM6 expression with TME and different cancer subtypes across pan‐gastrointestinal cancers. (A) Analysis of CEACAM6 correlation with immune cell infiltrations with multiple Deconvolution algorithms; (B) Differential CEACAM6 expression levels in MSI‐L, MSS and MSI‐H groups; (C) Differential CEACAM6 expression levels across immune infiltrate subtypes in pan‐gastrointestinal cancers.

To evaluate the role of CEACAM6 in immune regulation, we analyzed its associations with immune cell infiltration, stromal composition, and MSI across pan‐gastrointestinal cancers. CEACAM6 expression demonstrated consistent negative correlations with anti‐tumor immune components, including CD4 + Th1 cells, activated dendritic cells, NK cells, and M1 macrophages, while positively linked to neutrophil (Figure 4A). Furthermore, CEACAM6 expression was negatively associated with cytotoxicity scores, immune scores, and microenvironment scores in COAD and STAD, indicating a broader immunosuppressive impact (Figure 4A). MSI status analysis revealed that CEACAM6 expression was significantly higher in MSI‐Low group compared to MSI‐H group in COAD and STAD cohorts (Figure 4B). This suggests a potential link between CEACAM6 and immune evasion in low‐immunogenic tumors. To further dissect immune contexture, six immune subtypes (C1‐C6) were identified in pan‐gastrointestinal cancer cohort based on The Immune Landscape of Cancer. High CEACAM6 expression was enriched in the C1 (wound healing) subtype, characterized by Th2 bias, angiogenic gene activation, and high proliferation. Conversely, low CEACAM6 levels dominated the C2 (IFN‐γ dominant) subtype, marked by CD8 + T cell infiltration and M1 macrophage polarization (Figure 4C). These findings align with CEACAM6's role in shaping an immunosuppressive tumor microenvironment. In summary, CEACAM6 fosters an immunosuppressive tumor microenvironment, playing a vital role in the cancer‐immune crosstalk to facilitate immune escape.

**Figure 5:**
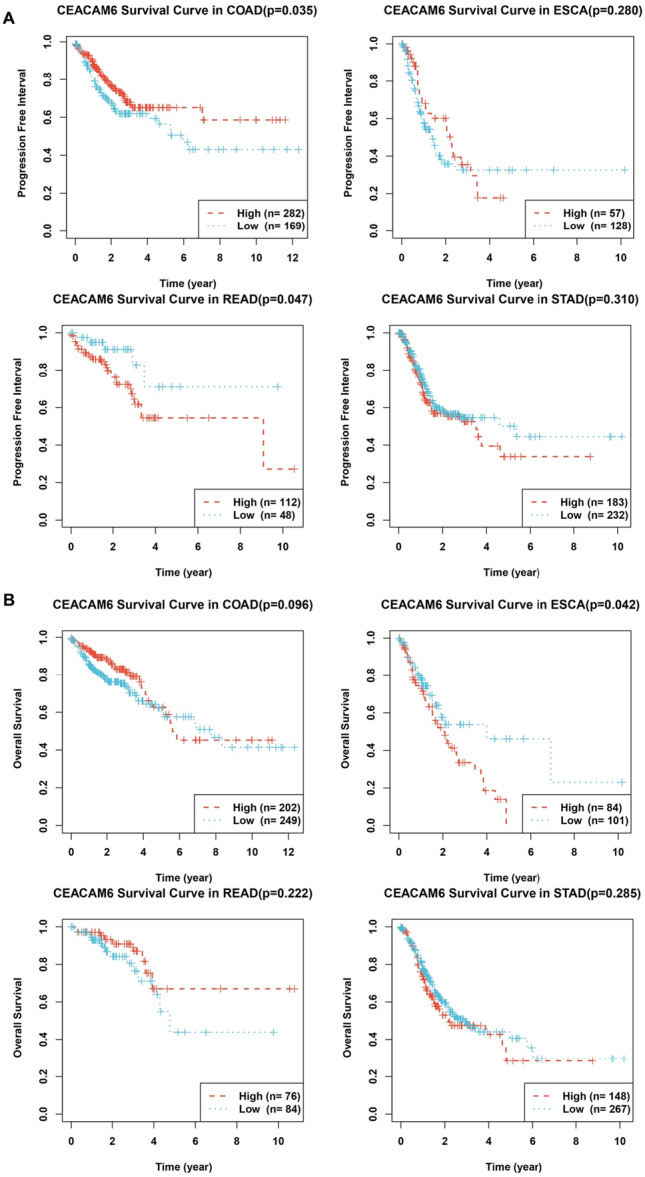
Kaplan–Meier survival curves for CEACAM6 expression in pan‐gastrointestinal cancers. (A) Kaplan‐Meier survival curves of PFS for different CEACAM6 expression in pan‐gastrointestinal cancers; (B) Kaplan‐Meier survival curves of OS for different CEACAM6 expression in pan‐gastrointestinal cancers.

Prognostic relevance was confirmed via Kaplan‐Meier analysis. High CEACAM6 expression correlated with poorer progression‐free survival (PFS) in COAD (*p *= 0.035) and READ (*p *= 0.047), as well as reduced overall survival (OS) in ESCA (*p *= 0.042) (Figure 5). Collectively, these data revealed that CEACAM6 indicated the poorer clinical outcomes across pan‐gastrointestinal malignancies.

### 3.5 Spatial Transcriptomic (ST) to Explore the Implication of CEACAM6

**Figure 6:**
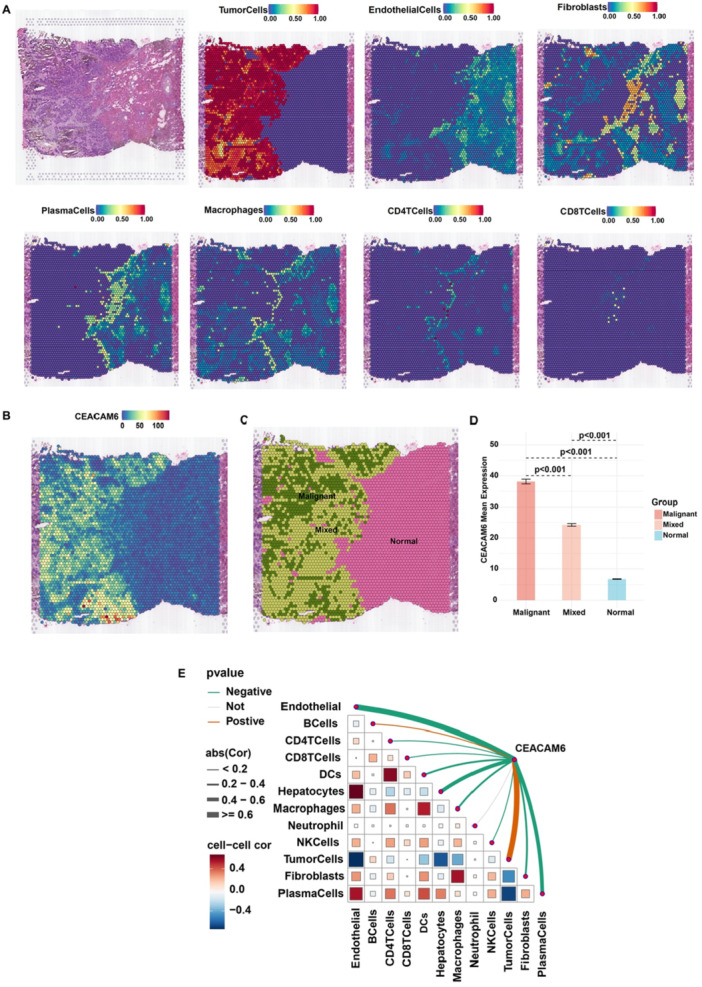
Expression of CEACAM6 in spatial transcriptomic of colorectal cancer with liver metastases. (A) Predicted cell types mapping on colorectal cancer tissue slice; (B) Spatial distribution of CEACAM6; (C) Spatial distribution of malignant region, mixed region and normal region; (D) The difference expression of CEACAM6 in malignant region, mixed region and normal region; (E) The correlation heatmap between CEACAM6 expression and different cell types.

To spatially resolve CEACAM6 expression within the tumor microenvironment, we performed 10x Visium spatial transcriptomic analysis of a colorectal cancer liver metastasis specimen (ST‐P1). Deconvolution analysis revealed a clear spatial association between high CEACAM6 expression and tumor cell clusters (Figure 6A–B). Quantitative analysis across distinct tissue regions demonstrated that CEACAM6 expression was significantly elevated in malignant areas compared to mixed (*p* < 0.001) and normal regions (*p* < 0.001; Figure 6C–D). Spatial correlation analysis further established CEACAM6's association with immune exclusion, showing significant negative correlations with plasma cells, macrophages, and dendritic cells, while maintaining strong positive correlation with malignant cells (Figure 6E). These spatial findings provide direct histological evidence that CEACAM6 is predominantly expressed in malignant epithelial cells and is associated with an immune‐suppressed niche in pan‐gastrointestinal cancer.

## 4 Discussion

Pan‐gastrointestinal malignancies, due to their aggressive nature, often present at advanced stages, resulting in high mortality rates. Over the past two decades, progress has been made in non‐surgical treatment strategies for these cancers. Specifically, the combination of immunotherapy and chemotherapy has become the standard first‐line treatment for advanced gastric cancer, demonstrating notable tumor regression and downstaging effects in the perioperative setting, particularly in patients with PD‐L1 CPS > 1 [33]. Additionally, immune checkpoint inhibitors, such as PD‐1 or PD‐L1 antibodies, have shown remarkable efficacy in solid malignancies with microsatellite instability‐high (MSI‐H), including MSI‐H colorectal cancer [34]. However, given the 1.4 million annual global deaths attributed to pan‐gastrointestinal cancers [1], there is still an urgent unmet medical need for new biomarkers to improve the diagnosis and treatment of these cancers. Previous studies often offered fragmented observations‐focusing on single cancer types or isolated cellular functions of CEACAM6. In this study, our integrated multi‐omics analysis demonstrates that CEACAM6 is robustly and consistently upregulated at the membrane of malignant cells across colorectal, gastric, and related gastrointestinal cancers, correlating with adverse survival and features of aggressive biology. Clinically, this molecular profile positions CEACAM6 as a versatile translational asset: (i) a high‐performance diagnostic and prognostic biomarker (ROC AUC > 0.9 in COAD and STAD; concordant IHC protein upregulation), supporting its inclusion in tissue and circulating‐RNA panels for earlier detection and risk stratification; (ii) a predictive marker candidate for microenvironment‐directed therapies given its association with immune‐excluded TMEs; and (iii) a tractable membrane target for precision therapeutics including ADC, radiolabeled antibodies, and engineered cell therapies, owing to its tumor‐restricted surface localization. Preclinical work supporting ADCs, CAR‐T constructs and CEACAM6‐directed radiotherapy underscores immediate paths toward first‐in‐human testing, while also arguing for companion diagnostics to select antigen‐positive patients and monitor on‐treatment target modulation. These findings paved the avenues for CEACAM6‐targeted therapies.

Mechanistically, CEACAM6 orchestrates a multifaceted oncogenic program by driving tumor cell survival, activating oncogenic and metastatic pathways, conferring acid‐resistance phenotypes, and facilitating lactylation‐dependent metabolic reprogramming, thereby underpinning chemoresistance and immunotherapy resistance. Previous study reported that CEACAM6 expression was significantly associated with acid resistance and that CEACAM6 was a biomarker for acid‐resistant clones [35]. These collective features provide a compelling rationale for combining CEACAM6‐directed agents with driver‐targeted therapies (e.g., EGFR/HER2 inhibitors) or metabolic modulators to counter adaptive resistance. Critically, CEACAM6 exerts its immunosuppressive function through homophilic and heterophilic interactions within the CEACAM family proteins (CEACAM1‐CEACAM6, CEACAM5‐CEACAM6 and CEACAM8‐CEACAM1) [36] [37]. Bolland, et al. reported that the cytoplasmic domain of CEACAM1 share similarity with the consensus sequence of the immunoreceptor tyrosine‐based inhibition motif (ITIM) in DT40 B cells [38]. The ITIM was the docking site for SHIP, SHP‐1, and SHP‐2. The dimeric state of CEACAM1 could mediate inhibitory signals by the cytoplasmic tail to the B cell receptor [38]. Besides, CEACAM1 is expressed on T cells following activation, and binding to extracellular ligands, such as CEACAM6, could lead to T cell downregulation [39]. These interactions between CEACAM6 and other CEACAMs could potentially inhibit the anti‐tumor activity of immune cells and facilitate immune evasion of malignancies. In this study, the link between CEACAM6 expression and the prevalence of distinct immune cell subsets, including a negative correlation with CD4 + Th1 cells, dendritic cells, M1 macrophages, and plasma cells, implies a potential role of CEACAM6 in modulating the TIME in pan‐gastrointestinal cancers. This is further supported by spatial transcriptomic data analysis. The mechanisms and findings described above establish a compelling mechanistic rationale for combining CEACAM6‐blocking agents with immune checkpoint inhibitors or for developing bispecific antibody formats, strategies designed to simultaneously redirect immune effector function and counteract tumor‐induced immune suppression.

This study characterizes CEACAM6 as a pivotal nexus coordinating both intrinsic tumorigenesis and extrinsic immune evasion in pan‐gastrointestinal cancers. CEACAM6 could drive malignant progression through oncogenic pathway activation while simultaneously sculpting an immunosuppressive microenvironment by excluding immunostimulatory cells. Collectively, these findings carried significant translational implications for CEACAM6 in pan‐gastrointestinal cancers. CEACAM6 has shown promise for both diagnostic and therapeutic applications in pan‐gastrointestinal cancers. Anand D. Jeyasekharan et al. reported that CEACAM6 could be up‐regulated by Helicobacter pylori CagA and was an early‐detection biomarker in gastric cancer [40]. A CEACAM6‐targeted fluorescent probe has been developed to enhance endoscopic detection of precancerous gastric lesions or early gastric cancer [13]. Additionally, a blood‐based assay incorporating CEACAM6 mRNA demonstrates potential for noninvasive early detection of colorectal neoplasia [41]. Moreover, the tumor‐specific membrane localization of CEACAM6 makes it an ideal drug delivery target for precision therapeutics. Recent studies have focused on the development of antibody‐drug conjugates (ADCs) or chimeric antigen receptor T (CAR‐T) cells targeting CEACAM6. The ADC could combine the specificity of monoclonal antibodies with the cytotoxic potential of chemotherapeutic agents, offering a targeted approach to eliminate cancer cells while sparing normal tissues. Our previous work developed a CEACAM6‐directed ADC, which demonstrated significant anti‐tumor efficacy and enhanced immune cell infiltration in patient‐derived xenograft models [42]. Machinaga, et al. designed a CEACAM6‐targeting ADC with a BET degrader payload, demonstrating efficacy in pancreatic cancer organoids and TME modulation [43]. CEACAM6‐targeted chimeric antigen receptor (CAR) T‐cell therapy constitutes another promising strategy. Substantial preclinical studies demonstrated CAR‐T cells targeting CEACAM6 exhibited robust antitumor efficacy across multiple gastrointestinal cancer models [44, 45]. Hardt, et al. performed an empirical screening of 371 cancer‐related antigens and identified CEACAM6 as the candidate target of CAR‐T cell therapy [44]. Furthermore, CEACAM6 has emerged as a viable target for radioisotope therapy, as evidenced by a CEACAM6‐targeting antibody conjugated to ^131^I exhibiting significant anti‐tumor activity [46]. These preclinical findings underscore CEACAM6's clinical potential across pan‐gastrointestinal malignancies. Nevertheless, it is worth pointing out that CEACAM6 functions as a “non‐oncogene addiction“ target due to its pleiotropic pro‐tumorigenic roles. Thus, combining CEACAM6‐targeted strategies with driver gene inhibition (e.g., EGFR/HER2) may yield a synergistic anti‐tumor effect and superior therapeutic outcomes.

This study opens several compelling avenues for future investigation. Firstly, CEACAM6's tumor‐specific membrane overexpression makes it an attractive target for next‐generation engineered immune cell therapies (CAR‐macrophages or CAR‐NKs). Meanwhile, CEACAM6 could also be a potential target of bispecific and multi‐specific antibodies [47]. Secondly, the membrane‐restricted localization of CEACAM6 may minimize the potential off‐target toxicity. A number of candidates for bispecific antibody formats include CEACAM6×HER2, CEACAM6×PD‐1, and CEACAM6×CD3, for which proof‐of‐concept studies are currently underway. Thirdly, another future direction is the mechanistic validation of CEACAM6's upstream regulators, notably the transcription factors KLF5 and SP3, utilizing CRISPR‐Cas9‐based knockout in relevant cell models. Lastly, investigating how CEACAM6 blockade modulates the TME represents a promising therapeutic avenue. CEACAM6 antibody has shown synergistic effects with TIM‐3 and PD‐1 blockade in augmenting T cell‐mediated tumor cytotoxicity [9]. The previous study also reported that CEACAM6 overexpression correlated with reduced infiltration of immune T cells during colorectal carcinogenesis [48]. It should be noted that CEACAM6 exhibits exclusive expression in primates, precluding direct investigation of its TME regulatory roles in conventional murine models. This species‐specific limitation necessitates alternative experimental approaches, such as patient‐derived organoids co‐cultured with peripheral blood mononuclear cells (PBMCs) to faithfully recapitulate CEACAM6‐mediated immunomodulation.

There were several limitations that are intrinsic to the type of data available and the analysis performed in this study. Firstly, while leveraging TCGA data provides valuable exploratory insights, the retrospective nature inherently limits causal inference and may introduce selection bias. Secondly, the identified transcription factors regulating CEACAM6 in this study require experimental validation. Accordingly, the authors plan to employ CRISPR‐Cas9 to knock out the identified transcription factors and then detect changes of CEACAM6 RNA and protein expression to verify the roles of the identified transcription factor. Thirdly, current limitations in ATAC‐seq bioinformatics tools constrain data interpretation [49]. Additionally, anal cancer was not included in this study, as RNA transcriptomic data for this cancer type are unavailable in public databases.

## 5 Conclusion

In summary, CEACAM6 exhibits high diagnostic accuracy and tumor‐specific overexpression in pan‐gastrointestinal cancers. CEACAM6 could promote angiogenesis/metastasis and suppress anti‐tumor immunity. Spatially localized in tumors with immune cell exclusion, CEACAM6 correlates with poor survival and immune‐excluded subtypes, positioning it as a therapeutic target in precision immunotherapy for pan‐gastrointestinal cancers.

## Author Contributions

**Liying Jin:** data curation, investigation, methodology. **Changjuan Tao:** formal analysis; methodology, validation, visualization.

## Ethics Statement

This study was approved by the Institutional Animal Care and Use Committee (IACUC) of Zhejiang Cancer Hospital (No. IRB‐2024‐659).

## Conflicts of Interest

The authors declare no conflicts of interest.

## Data Availability

All data relevant to the study are included in the article.
